# Bronchioalveolar morphogenesis of human bronchial epithelial cells depending upon hepatocyte growth factor

**DOI:** 10.1111/jcmm.12672

**Published:** 2015-09-28

**Authors:** Takashi Kato, Kiyomasa Oka, Toshikazu Nakamura, Akihiko Ito

**Affiliations:** ^1^ Department of PathologyFaculty of MedicineKinki UniversityOsaka‐SayamaOsakaJapan; ^2^ Department of PharmacologyFaculty of MedicineKinki UniversityOsaka‐SayamaOsakaJapan; ^3^ Research & DevelopmentNeurogen Inc.IbarakiOsakaJapan

**Keywords:** alveolar regeneration, bronchial cell, hepatocyte growth factor, lung adenocarcinoma histogenesis, three‐dimensional culture

## Abstract

Lung alveolar regeneration occurs in adult human lungs as a result of proliferation, differentiation and alveolar morphogenesis of stem cells. It is increasingly being believed that bronchial epithelial cells (BECs) have a potential as stem cells, because they are potent to differentiate into multiple central and peripheral lung cell types in three‐dimensional (3D) cultures, and they develop multiple foci with well‐differentiated histogenesis after transformed into neoplastic cells. In this study, we investigated morphogenic abilities of HBE135 human BECs immortalized by E6/E7 oncogene in 3D cultures. When HBE135 cells were cultured alone or co‐cultured with endothelial cells, the cells formed spherical colonies without branching. However, in co‐culture with lung fibroblast MRC‐9 cells, HBE135 cells formed colonies with bronchioalveolar‐like complex branching, suggesting that MRC‐9‐derived soluble factor(s) are responsible for the branching formation. MRC‐9 cells, not endothelial cells, were found to highly express hepatocyte growth factor (HGF), a soluble molecule involved in liver and kidney regeneration. An anti‐HGF neutralizing antibody severely suppressed the complex branching formation, but addition of HGF could not sufficiently compensate the morphogenic effects of MRC‐9 cells, suggesting that MCR‐9‐derived HGF was necessary but insufficient for the bronchioalveolar structure formation. Immunohistochemistry revealed that Met, a cognate receptor for HGF, was highly expressed and phosphorylated in neoplastic BECs from lung adenocarcinomas with well‐differentiated, not poorly differentiated, histogenesis. These results are consistent with the notion that BECs have an aspect of stem cells. This aspect appears to become manifest through HGF–Met signalling pathway activation.

## Introduction

Lung cells are generally controlled in a strict spatiotemporal fashion by several soluble factors for proper organ development 1. Human normal lungs have the capability to initiate repair and regeneration following any insult and involve cell proliferation, differentiation and morphogenesis, but the detailed mechanisms are largely unknown. Importantly, a recent report indicates that an adult human lung can regrow as demonstrated by an increase in vital capacity, enlargement of the remaining left lung and increased alveolar numbers in a patient who underwent right‐sided pneumonectomy 2. Cell turnover is normally slow in the alveolar region of the adult lung. However, after experimental injuries that damage and kill alveolar cells and/or expose them to oxidative stress, there is rapid proliferation of the surviving cells and activation of repair mechanisms. Upon failure of these processes, the pathological response varies, from emphysema to extensive interstitial fibrosis. To develop strategies for regeneration of the adult lung, we need to deepen our understanding of normal lung development and growth, and identify active developmental signalling systems that might be promising targets for therapeutic approaches.

Multipotent progenitor cells exist within the adult murine lung, which are postulated to give rise to all of the stem/progenitor cells 3. There is now overwhelming evidence that basal cells are multipotent stem cells in the tracheobronchial region, which can both self‐renew and give rise to ciliated and secretory lineages during post‐natal growth, a steady‐state and repair following damage to the epithelium. *In vitro* culture techniques including air–liquid interface and three‐dimensional (3D) clonal cultures enable analysis of the potential of single cells to self‐renew and differentiate into ciliated and secretory cells 4, 5. Moreover, human bronchial epithelial cells (BECs) display characteristics of multipotent stem cells of the lung 6. When cultured in 3D systems, subtle changes in the microenvironment result in unique responses including the ability of human BECs to differentiate into multiple central and peripheral lung cell types. Therefore, the adult human lung contains a multipotent progenitor cell type with a differentiation potential that is primarily dictated by the microenvironment. Interestingly, human BECs often retain their morphogenic ability after they are transformed into neoplastic cells, as demonstrated by the fact that the resulting tumours generally have various histological components, each of which is highly morphogenic, and thereby are diagnosed as adenocarcinoma mixed subtypes 7. Although molecular mechanisms for lung adenocarcinoma histogenesis have not yet been studied intensively, this morphogenic ability displayed by neoplastic epithelial cells may reflect the nature of human BECs as stem cells.

Hepatocyte growth factor (HGF) acts as a key regulator in various biological events including liver and kidney regeneration, suggesting that HGF has a morphogenic action 8. In fact, when kidney epithelial Madin Darby Canine Kidney (MDCK) cells are grown in collagen gels containing HGF, they form branching tubules instead of spherical cysts 9. HGF is also suggested to contribute to lung regeneration. Plasma and local HGF levels increase in response to lung injury under pathological conditions, and HGF exerts mitogenic and anti‐apoptotic effects on lung epithelial cells 10. We previously suggested that HGF might act as a potent multifunctional pulmotropic factor that induces the formation of alveolar networks from destroyed alveolar cells in injured lung tissues 11, 12. Although selective deletion of the *c‐met* gene encoding a cognate receptor for HGF in respiratory epithelium leads to malformation of alveolar septae 13, it remains elusive whether HGF can contribute to bronchioalveolar morphogenesis during lung regeneration.

HBE135‐E6/E7 (here, called shortly HBE135) cells are a human BEC line immortalized by E6/E7 oncogene but not tumourigenic, and have not been shown to display any morphogenic characteristics of stem cells 14, 15. In the present study, we first examined morphogenic potentials of HBE135 cells in 3D cultures, and found that the cells were able to form bronchioalveolar structures in the co‐presence of lung fibroblasts. Next, we examined roles of the fibroblasts, and found that fibroblast‐derived HGF was necessary for HBE135 cells to form bronchioalveolar structures. Finally, we examined the relevance for this action of HGF in an *in vivo* setting. Immunohistochemistry provided evidence that signalling pathways mediated by HGF and its receptor Met were activated in well‐differentiated adenocarcinomas where neoplastic BECs were highly morphogenic. These results not only provide new evidence that BECs have an aspect of stem cells but also suggest that induction of this aspect largely depends upon HGF–Met signalling pathway activation.

## Materials and methods

### Cell culture

HBE135 cells were obtained from the American Type Culture Collection (Manassas, VA, USA). The cells were cultured as manufacturer's instruction. Human umbilical vein endothelial cells (HUVECs) and human microvascular endothelial cells (HMVECs) were purchased from Lonza (Allendale, NJ, USA) and grown in MCDB131 medium supplemented with 5% foetal bovine serum, 2 mM L‐glutamine and 20 ng/ml basic fibroblast growth factor (FGF). MRC‐9 cells (human lung fibroblasts) and SF4‐1 cells (human skin fibroblasts) were cultured in DMEM supplemented with 10% foetal bovine serum.

For 3D culture experiments, we used growth factor‐reduced rBM (Matrigel; BD Biosciences, Bedford, MA, USA). Cells were mixed with 100 or 50 μl Matrigel in its liquid state, plated onto 8‐ or 16‐well chamber slides respectively, and allowed to gelatinize at 37°C for an hour before adding 1 ml of culture medium. For culture experiments, each cell was seeded into the Matrigel to ensure clonal growth and reduces the aggregation of epithelial cells. Afterwards, the medium was changed every 3 days. A spherical colony was defined as a single sphere. A budding colony was defined as that with multiple spheres. A branching colony was defined as that with spherical parts and their ramification parts. A complex branching colony was defined as that with some branching parts. We counted the total and branching colonies one by one under a phase‐contrast microscope. We evaluated the total colonies in eight‐well dishes for each experiment.

### Immunofluorescence analysis

Three‐dimensional cultures were fixed with ice‐cold 4% paraformaldehyde in PBS at 4°C for 30 min. After permeabilization with 0.3% Triton X‐100 in PBS for 10 min., the cells were blocked with 5% normal goat serum in PBS for 1 hr at room temperature. Then, the cells were incubated with primary anti‐E‐cadherin (36/E‐cadherin; BD Biosciences, San Jose, CA, USA) and anti‐surfactant protein‐C pro‐peptide (pro‐SP‐C; AB3786; Chemicon International, Temecula, CA, USA) and anti‐vimentin (Clone V9; DAKO, Glostrup, Denmark) antibodies at 4°C overnight. After washing with PBS, the cells were incubated with Alexa 488‐conjugated IgG (1:600; Invitrogen, Carlsbad, CA, USA) as secondary antibodies and the nuclear dye propidium iodide for 30 min. at room temperature, and observed as described previously 16. For a 5‐bromo‐2′‐deoxyuridine (BrdU) assay, HBE135 cell colonies cultured for 9 days were incubated for a further 3 days with 10 μM BrdU (Nacalai Tesque, Kyoto, Japan). The colonies were fixed in 4% paraformaldehyde, and BrdU incorporation was assayed by immunofluorescence using an anti‐BrdU antibody (OBT0030; AbD Serotec, Oxford, UK).

### Western blot analysis

Cells were lysed on ice with lysis buffer [50 mM Tris‐HCl (pH 7.5), 150 mM NaCl, 1% Triton X‐100, 5 mM EDTA, protease inhibitor cocktail, 2 mM Na_3_VO_4_ and 50 mM NaF]. The lysates were immunoblotted as described previously 16. Anti‐phospho‐Met (1234/1235) IgG [D26; Cell Signaling Technology (CST), Beverly, MA, USA], anti‐Met IgG (C‐12; Santa Cruz Biotechnology, Dallas, TX, USA), anti‐phospho‐ERK1/2 IgG (E10; CST), anti‐ERK1/2 IgG (137F5; CST), anti‐phospho‐ AKT IgG (#9271; CST) and anti‐AKT IgG (#9272; CST) antibodies were used respectively.

### Quantitative RT‐PCR

Total RNA was extracted from cells using Trizol (Invitrogen). The quantitative RT‐PCR was performed as described previously 16. Expression was normalized to *GAPDH* mRNA levels. The specific primers used in this study are described in Table S1.

### Immunohistochemical analysis of human lung cancer specimens

Human lung cancer specimens (mixed subtype) were obtained from patients diagnosed with lung cancer, who underwent tumour removal surgery at Kinki University Hospital (Osakasayama, Osaka, Japan). Tumour specimens were fixed with 10% buffered formalin, embedded in paraffin and cut into 4‐μm sections. The sections were subjected to immunohistochemical staining for Met (C12; Santa Cruz Biotechnology) and phospho‐Met (Tyr1234/1235, D26; CST). The Ethics Committee of Kinki University approved the experimental protocol and waived the need for written informed consent (24–071). Met immunoreactivities were evaluated as follows in accordance with the methods with slight modification 17: 0, complete absence of staining or only focal weak staining; 1+, weak to moderate staining in less than 40% of cancer cells; 2+, weak to moderate staining in at least 40% of cancer cells; 3+, strong staining in at least 10% of cancer cells, among the species with weak to moderate staining in at least 40% of cancer cells. Weak to moderate staining was defined as staining similar to or weaker than that of normal alveolar epithelium; strong staining was defined as staining clearly more intense than that of normal alveolar epithelium. The specimens graded as Met high (2+ or 3+) were positive and counted in well or poorly differentiated cancers. Immunoreactivities for phospho‐Met were evaluated as either positive or negative, with a cut‐off value of 5% of positively stained cancer cells 17.

### Statistical analysis

The Student's *t*‐test was performed to evaluate the significant differences in number of colonies. The chi‐squared test was used to assess the significant differences in histological positivity of Met and phospho‐Met expression in lung adenocarcinoma.

## Results

### Mesenchymal cells play various roles in branching morphogenesis of airway epithelial cells

**Figure 1:**
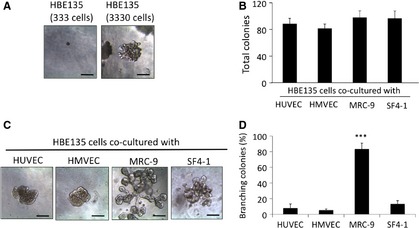
Effects of mesenchymal cells on colony formation in 3D culture. (**A**) Phase‐contrast images of HBE135 cells cultured alone in rBM. HBE135 cells did not form colonies at a low cell density (333 cells in 100 μl rBM, left panel), but formed spherical colonies at a high cell density (3330 cells in 100 μl rBM, right panel). (**B**–**D**) HBE135 cells were cultured in rBM with various types of mesenchymal cells; scale bars 100 μm. (**B**) Numbers of colonies formed by HBE135 cells in coculture with mesenchymal cells (3 × 10^3^ cells/ml) in rBM. Each value represents the mean ± S.D. (*n* = 4). (**C**) Phase‐contrast images of HBE135 cell colonies co‐cultured with mesenchymal cells [human umbilical vein endothelial cells (HUVECs), human microvascular endothelial cells (HMVECs), MRC‐9 cells and SF4‐1 cells]; scale bars 100 μm. (**D**) Numbers of colonies formed by of HBE135 cells with a complex branching structure. Each value represents the mean ± S.D. (*n* = 4); scale bars: 100 μm. ***P < 0.001

Franzdóttir *et al*. reported that the human BEC line VA‐10 forms polarized spherical colonies when grown in 3D culture 18. We first cultured HBE135 cells alone in rBM at the same concentration (333 cells per 100 μl rBM), but the HBE135 cells did not form colonies (Fig. 1A). However, when cultured at a high cell density (3330 cells per 100 μl rBM), HBE135 cells formed spherical colonies (Fig. 1A), although these colonies were not accompanied by a branching structure. VA10 cells form a branching structure in rBM when co‐cultured with HUVECs 18, but we did not observe branching colonies of HBE135 cells in co‐culture with HUVECs (Fig. 1B). We thus investigated whether other mesenchymal cell types affect epithelial branching morphogenesis. Human microvascular endothelial cells, human skin fibroblasts (SF4‐1 cells) and human lung fibroblasts (MRC‐9 cells) were also co‐cultured with HBE135 cells. A total of 333 cells of HBE135 were co‐cultured with mesenchymal cells at 3 × 10^3^ cells/ml in Matrigel respectively. All of the examined mesenchymal cell types had a similar potential to induce formation of epithelial colonies in 3D culture (Fig. 1B). HBE135 cells formed colonies in co‐culture with endothelial cells (HUVECs and HMVECs), which exhibited mostly spherical or budding morphology but not branching (Fig. 1C). In contrast, MRC‐9 cells induced a complex branching structure of HBE135 cells (Fig. 1C). Some colonies were branching in co‐culture with SF4‐1 cells. However, in co‐culture with MRC‐9 cells, 83.3 ± 7.6% of the colonies showed complex branching (Fig. 1D). These mesenchymal cell types cultured alone in rBM showed no remarkable changes (*e.g*. proliferation) for the experimental period (data not shown). Therefore, MRC‐9 cells underwent morphogenesis to form a bronchioalveolar structure of HBE135 cells in 3D culture.

### Characterization of co‐culture conditions with lung fibroblasts and the bronchioalveolar structure of HBE135 cells by immunofluorescence

**Figure 2:**
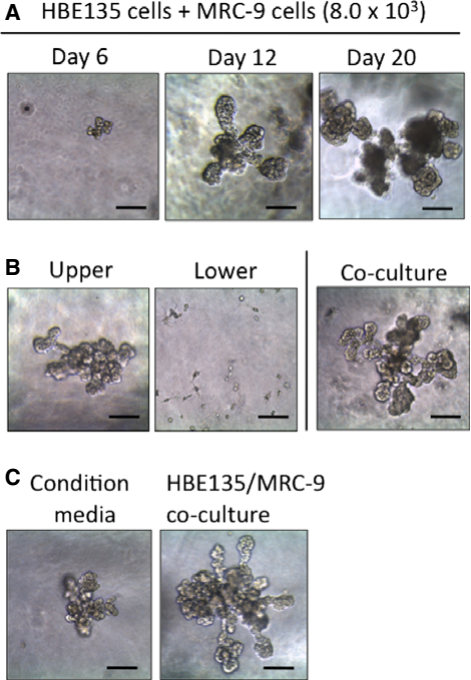
Characterization of HBE135 cell colonies co‐cultured with MRC‐9 cells in rBM. (**A**) Phase‐contrast images of HBE135 cell colonies (333 cells in 100 μl rBM) co‐cultured with MRC‐9 cells (8 × 10^3^ cells) at days 6, 12 and 20. (**B**) Phase‐contrast images of HBE135 cell colonies (left) separated from MRC‐9 cells (centre) cultured at 20 days. (**C**) Induction of the branching formation of HBE135 cells by conditioned media (left). (**B** and **C**) Branching formation of HBE135 cells co‐cultured with MRC‐9 cells as a positive control (right); scale bars: 100 μm.

To examine branching morphogenesis *in vitro*, we analysed the branching process of HBE135 cells in co‐culture with MRC‐9 cells. Early colonies were observed after 6 days of culture, and the branching structure became gradually more complex by 12 days (Fig. 2A). These bronchioalveolar structures had grown by 20 days (Fig. 2A). Further culture of the colonies did not result in any characteristic formations. To investigate whether this morphogenic activity was induced by cytokines released from fibroblasts, HBE135 cells were suspended in rBM on top of a preformed gel layer containing MRC‐9 cells. A cell‐free gel layer was interposed between the two layers of HBE135 and MRC‐9 cells to avoid contact of the two layers. As a result, HBE135 cells exhibited branching morphogenesis in the upper layer (Fig. 2B). We did not observe movement of the MRC‐9 cells in the lower layer to the upper layer. Subsequently, HBE135 cells were incubated in conditioned medium from cultures with preformed complex branching structures. A small number of colonies were formed under this condition (3.2 ± 0.7 colonies/100 μl rBM). These colonies had a symptom of branching, but were not equivalent to those under the co‐culture condition (Fig. 2C).

**Figure 3:**
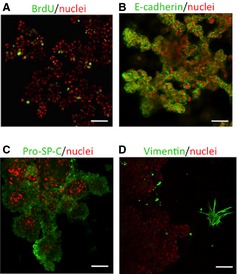
Proliferating cells in various regions of the branching epithelium, and expression of alveolar epithelial markers in the branching structure of HBE135 cells co‐cultured with MRC‐9 cells in Matrigel at 16 days. Confocal images of immunofluorescently labelled structures in rBM. 5‐bromo‐2′‐deoxyuridine (BrdU) (**A**), E‐cadherin (**B**), pro‐SP‐C (**C**) and vimentin (**D**) are green. Nuclear staining with propidium iodide (PI) is shown as red; scale bars: 50 μm.

**Figure 4:**
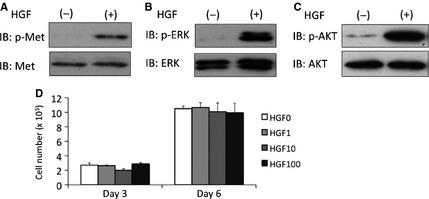
Hepatocyte growth factor (HGF) has no mitogenic activity in HBE135 cells. (**A** and **B**) Immunoblot analysis showing HGF reactivity in HBE135 cells. (**A**) Changes in phosphorylated Met (top) and total Met (bottom) protein levels were detected using anti‐phosphorylated Met (Tyr1234/1235) and ‐total Met antibodies. (**B**) Changes in phosphorylated ERK1/2 (top) and total ERK1/2 (bottom) protein levels were detected using anti‐phospho‐ERK1/2 (Thr202/Tyr204) and ‐total ERK antibodies. (**C**) Changes in phosphorylated AKT (top) and total AKT (bottom) protein levels were detected using anti‐phospho‐AKT (Ser473) and ‐total AKT antibodies. (**D**) Effects of HGF on cell proliferation. Quantification of HBE135 cells after 3 and 6 days of culture in medium supplemented with rhHGF (1, 10 and 100 ng/ml). Each value represents the mean ± S.D. (*n* = 4).

To characterize the complex branching, we first identified the proliferating cells in the branching structure by immunofluorescence. We found BrdU‐positive cells in various regions of the complex branching structure (Fig. 3A). We next examined the expression of cell markers in the branching structures. As an epithelial cell marker, E‐cadherin was stained at 12 days of culture. E‐cadherin was clearly seen at plasma membrane in the branching structure, especially at the distal area (Fig. 3B and Fig. S1). Furthermore, expression of pro‐SP‐C, a marker of ATII cells, was observed in the branching structure (Fig. 3C). We also examined aquaporin‐5 (AQP‐5), an ATI cell‐specific protein, but did not observe its expression in the branching structure (data not shown). Furthermore, we confirmed that the branching structure did not express vimentin as a mesenchymal marker (Fig. 4D). The Met in this branching structure of HBE135 cells was not phosphorylated under 3D culture (Fig. S2A). It is possible that HGF–Met signalling is the early/initiation event for morphogen, and that epidermal growth factor (EGF) and/or other factors are involved in the middle or late events. On the other hand, Met phosphorylation was reduced after long‐time exposure of HGF in HBE135 cells under monolayer culture (Fig. S2B), and thus, Met phosphorylation might be inhibited by negative feedback mechanisms.

### HGF is highly expressed in MRC‐9 cells and has no mitogenic effects on HBE135 cells

To identify the soluble factors responsible for the complex branching of MRC‐9 cells, we investigated the mRNA levels of growth factors that have been reported to accelerate alveolarization. FGF1 and VEGF‐A mRNA expression in SF4‐1 cells were higher than that in MRC‐9 cells, and heparin‐binding EGF (HB‐EGF) mRNA expression was higher in HUVECs (Table 1). However, HGF expression was dramatically higher in MRC‐9 cells compared with that in the other mesenchymal cell types. Thus, HGF might play important roles in the bronchioalveolar formation. To confirm whether MRC‐9 cells highly express HGF protein, 24‐hr conditioned media of the mesenchymal cells were analysed by ELISA. Although HGF protein was not found in the conditioned media of HUVECs and HMVECs, 15.1 ± 5.0 ng/ml HGF protein was detected in the culture medium of MRC‐9 cells. SF4‐1 cells secreted 1.34 ± 0.65 ng/ml HGF in 24 hrs of culture. We also examined the mRNA expression of another cytokine FGF10, which is dynamically localized in the mesenchyme surrounding prospective epithelial buds and has been shown *in vitro* to act as a chemoattractant for epithelial cells 19, 20, 21. However, we did not detect FGF10 mRNA in MRC‐9 cells.

**Table 1:** Expression of cytokines that are closely related with alveolarization

	FGF1	FGF7	HGF	HB‐EGF	VEGF‐A
HBE135 cells	0.24 ± 0.17	0.06 ± 0.03	N.D.	1.85 ± 0.29	2.20 ± 0.59
HUVECs	0.05 ± 0.02	0.06 ± 0.02	N.D.	2.19 ± 1.07	0.59 ± 0.29
SF4‐1 cells	10.82 ± 3.52	0.79 ± 0.24	0.05 ± 0.03	1.03 ± 0.61	4.53 ± 1.14
MRC‐9 cells	1.00	1.00	1.00	1.00	1.00

Normalized by GAPDH. Compared with the expression of MRC‐9 cells.

N.D.: not detected; HGF: hepatocyte growth factor; HB‐EGF: heparin‐binding epidermal growth factor; FGF: fibroblast growth factor.

### HGF is required but insufficient for the formation of bronchioalveolar‐like structures

We found that HBE135 cells expressed reactive Met, a receptor for HGF, by immunoblotting (Fig. 4A), and HGF treatment induced ERK1/2 (Fig. 4B) and AKT (Fig. 4C) phosphorylation in HBE135 cells under monolayer culture. HBE135 cells could thus respond to HGF. However, HGF had no effect on the proliferation of HBE135 cells (Fig. 4D).

**Figure 5:**
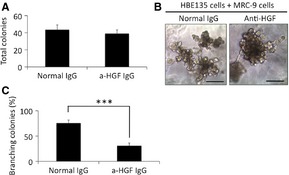
Hepatocyte growth factor (HGF) plays essential roles in branching morphogenesis of HBE135 cells. (**A**) Numbers of total colonies of HBE135 cells cultured in rBM with normal IgG and an anti‐HGF antibody. Each value represents the mean ± S.D. (*n* = 6). (**B**) Phase‐contrast images of HBE cell colonies treated with normal IgG and the anti‐HGF antibody for 16 days; scale bars: 100 μm. (**C**) Numbers of HBE135 cell colonies with complex branching in rBM containing the anti‐HGF antibody. Each value represents the mean ± S.D. (*n* = 6). ***P < 0.001

**Figure 6:**
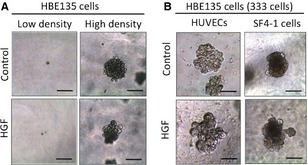
Hepatocyte growth factor (HGF) is insufficient to induce branching morphogenesis of HBE135 cells. (**A**) Phase‐contrast images of HBE135 cells treated with 10 ng/ml HGF in 3D culture at a low cell density and high cell density. (**B**) Phase‐contrast images of HBE135 cell colonies treated with or without rhHGF (10 ng/ml) in co‐culture with HUVECs and SF4‐1 fibroblasts. HBE135 cells were seeded at a low cell density; scale bars: 100 μm.

Next, to examine the role of the HGF–Met pathway in the branching formation of HBE135 cells, we used a neutralizing anti‐HGF antibody in 3D culture. In preliminary experiments, we confirmed blockade of HGF signalling by the anti‐HGF antibody at 10 μg/ml. The anti‐HGF antibody was added at the beginning of HBE135/MRC‐9 cell co‐cultures and could not prevent the colony formation (normal IgG: 43.6 ± 5.5 colonies *versus* anti‐HGF IgG: 38.2 ± 4.4 colonies; Fig. 5A). However, the anti‐HGF antibody inhibited the complex branching formation in 3D culture (Fig. 5B). Complex branching was formed by 75.6% of colonies in cultures with normal IgG, whereas only 31.1% of colonies formed complex branching in cultures with the anti‐HGF antibody (Fig. 5C). These results indicated that the HGF–Met system was important for the complex morphology in 3D cultures of HBE135 cells. Moreover, to verify whether HGF is sufficient for complex branching formation of HBE135 cells, we added recombinant human HGF (rhHGF) to 3D cultures of HBE135 cells alone. As shown in Figure 6A, we did not observe complex branching in these 3D cultures. We also added rhHGF to co‐cultures of HBE135 cells with HUVECs and SF4‐1 cells, but did not observe sufficient morphogenic activity for complex branching (Fig. 6B). These results suggested that MRC‐9 cells secrete factors other than HGF to induce complex branching formation.

### Possible involvement of HGF signalling pathway in human lung tumour histogenesis

**Figure 7:**
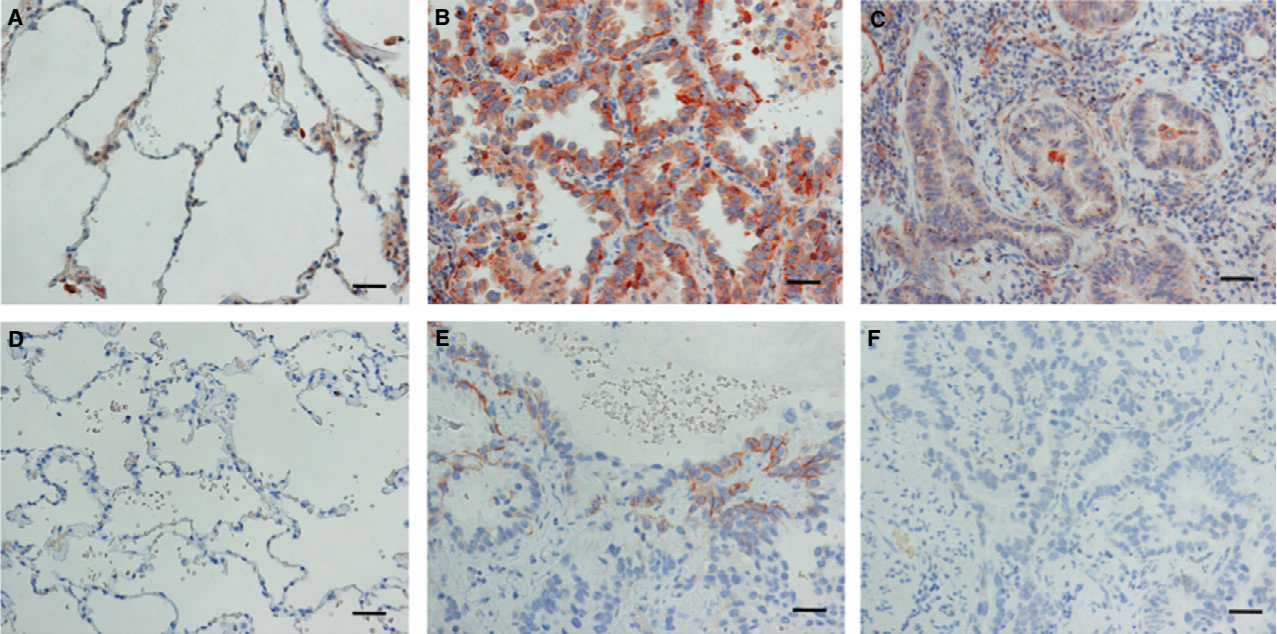
Expression of Met (**A**–**C**) and phospho‐Met (**D**–**F**) in normal and cancerous lung tissues. (**A**) Met expression was positive in ATII cells of normal tissues. (**B** and **C**) Met was strongly positive in the membranes of well‐differentiated cancer cells (**B**) and positive, but attenuated, in poorly differentiated cancer cells (**C**). (**D**) Phospho‐Met expression was negative in normal lung tissue. (**E** and **F**) Phospho‐Met was positive in the membranes of well‐differentiated cancer cells (**E**), but negative in poorly differentiated cancer cells (**F**); scale bars: 20 μm.

We considered that this complex branching formation was similar to lung adenocarcinoma, especially the well‐differentiated subtype. The HGF–Met pathway also plays an important role in tumourigenesis by promoting cell proliferation, survival, scattering, motility, migration, invasion and metastasis 22. To examine whether the complex branching structure was a useful model to study the molecular mechanisms of lung adenocarcinoma, we verified expression of Met in lung adenocarcinoma according to the degree of differentiation. Adenocarcinoma was selected from the mixed subtype as the histological diagnosis. As shown in Figure 7A, Met expression was found in normal ATII cells, but weak Met expression was found in other normal regions. Met expression was increased in well‐differentiated cancer cells, especially in membranous regions (Fig. 7B), and was relatively weak in poorly differentiated cancer cells (Fig. 7C). Histological quantification analysis revealed that Met signals were detected in 15 cases out of 35 well‐differentiated cancers and in 11 cases out of 35 poorly differentiated cancers, and thus, Met expression was not significantly correlated with the grade of differentiation (*P* = 0.32). As HGF exerts various effects *via* tyrosine phosphorylation (phospho‐Met), we examined phospho‐Met in lung adenocarcinoma. Phospho‐Met was not observed in normal regions of the lung (Fig. 7D). In contrast, phospho‐Met was positive in well‐differentiated cancer cells (Fig. 7E), but phospho‐Met signals were not detected in poorly differentiated cancer cells (Fig. 7F). Furthermore, Met phosphorylation could be found in almost half (*n* = 16) of 35 well‐differentiated cancers, but in only four tumours out of poorly differentiated cancers. This thus shows the significant increases in well‐differentiated cancers (*P* < 0.01). Therefore, HGF–Met signalling might play important roles as a morphogen in some well‐differentiated types of lung adenocarcinoma.

## Discussion

In the present study, we found that HBE135 cells had a potential to form bronchioalveolar structures in 3D cultures, and that this morphogenic process largely depended upon HGF. Although HGF appeared to actually act on HBE135 cells, it did not show a mitogenic activity in these cells. HGF might perform various roles, such as induction of mitogenesis or morphogenesis, according to the target cells, and play important roles in the morphogenesis of stem cells. However, HGF alone did not induce epithelial morphogenesis of HBE135 cells in 3D culture. In addition to HGF, MRC‐9 cells might secrete certain types of soluble factor(s) that are essential for the complex epithelial branching. In mesenchyme‐free culture of the foetal lung, HGF alone does not induce epithelial morphogenesis, but HGF together with FGF1 or FGF7 induce more extensive epithelial branching than that observed by treatment with FGF1 or FGF7 alone 23. SF4‐1 cells highly express FGF1 and FGF7 mRNA, but recombinant HGF could not induce the complex branching structure in the co‐culture system using SF4‐1 cells. Thus, it would be interesting to investigate the other factors secreted by MRC‐9 cells, which are responsible for the complex branching structure.

In the lung, an extensive pulmonary vascular network runs parallel to the airway and surrounds the intricate alveolar structure lined by respiratory epithelial cells. The close association between the two tissue compartments is essential for their functional coupling that is required for respiration. To generate alveolar structures, the vasculature and epithelium develop in a highly mutual‐dependent fashion. However, HBE135 cells did not form the complex branching structure in co‐culture with endothelial cells such as HUVECs and pulmonary HMVECs. As a possible reason, fibroblasts exert their morphogenic activity at an early‐phase, and then pulmonary endothelial cells might induce more differentiated bronchioalveolar structures. Because we did not observe expression of the ATI cell‐specific protein AQP‐5 in HBE135 cells, it would be interesting to verify whether HBE135 cell colonies with complex branching structures express AQP‐5 when co‐cultured with certain types of endothelial cells. However, VA10 cells form a complex branching structure in co‐culture with HUVECs 18. Although this discrepancy is not well understood, VA10 and HBE135 cells might be different cell types of the bronchiole or they might respond differently to the amount of HGF expressed by HUVECs.

After injury, lung stem cells must give rise to an appropriate number of differentiated progeny to achieve homoeostasis and restore the functional organ. The behaviour of epithelial stem cells is regulated by external signals from the microenvironment or niche in which stem cells reside. These signals include growth factors and other stem cell regulatory factors secreted by niche cells that can be a wide variety of differentiated cell types, including fibroblasts, smooth muscle cells, endothelial cells and neurons, as well as neighbouring stem cell progeny 24. HBE135 cells did not form any colonies in a collagen gel and required rBM to form colonies. In addition, conditioned medium from cultures with preformed complex branching had some morphogenic effects, but there was little colony formation (1–3 colonies/50 μl rBM). Therefore, soluble factors are insufficient for HBE135 cells to form colonies and a certain number of cells are required for proliferation in 3D culture. A specific microenvironment, such as matrix constitutes and cell–cell interactions, is required for HBE135 cells to form colonies. We will in detail examine the microenvironment or niche for branching morphology of BEC cells. In contrast, RLE‐6TN cells, a model of ATII cells, formed spherical colonies in rBM but did not exhibit the complex branching. It is noteworthy that, for the complex branching structure, tissue‐specific stem cells were required in 3D culture. Some important and unresolved problems exist, such as the complex branching had the characteristics of stem cells. Some reports suggest that the stem cells are c‐kit‐positive cells or bronchioalveolar stem cells, which coexpress the Clara cell marker Clara Cell secretary protein (CCSP) and ATII cell marker SP‐C in the lungs, especially involving repair/regeneration after respiratory damage 25, 26. We analysed the immunofluorescence studies and observed these protein‐positive cells at the rim of the branching structures (Fig. S3), suggesting that they are a kind of stem cell. However, further studies are needed to draw a conclusion on this issue.

We provided evidence that HGF–Met signalling pathway was activated in neoplastic histogenesis of BECs as exemplified by lung adenocarcinoma. Among lung adenocarcinomas, intermediate to high levels of Met correlates with a greater degree of tumour differentiation 27. Nakamura *et al*. immunohistochemically investigated HGF, Met and phospho‐Met (Tyr1235) in resected lung adenocarcinomas 17. They reported a significant association of phospho‐Met expression with tumour differentiation but not the pathological stage, lymph node metastasis or survival. In particular, phospho‐Met was correlated with papillary histology 17. As a possible reason, Met activation may require cell–cell or cell–matrix adhesions, and thus may be less efficient in poorly differentiated tumours 28. The HGF–Met system is involved in branching tubulogenesis of the lung 22 and other origins 28. In the present study, we found that the HGF–Met pathway induces HBE135 cells into a highly morphogenic, bronchioalveolar structure in 3D culture. Therefore, the complex branching formation of HBE135 cells may be a useful model to analyse the effects of Met activation on the well‐differentiated histogenesis of lung adenocarcinoma.

In conclusion, we presented a new 3D culture model to verify whether human BECs intrinsically have characteristics of pulmonary stem cells. In future studies, we intend to explore the more precise molecular mechanisms of bronchioalveolar morphogenesis and neoplastic histogenesis of human BECs by analysing this unique model with complex branching structures.

## Disclosure

The authors have no conflicts of interest.

## Supporting information


**Figure S1** Higher magnification of image for E‐cadherin in the branching structure co‐cultured with MRC‐9 cells in Matrigel. White arrows indicate that the E‐cadherin was stained stronger at cell–cell junctions; scale bars: 20 μm.

**Figure S2** (A) Met of the branching structure was not phosphorylated in 3D co‐culture with MRC‐9 cells. Immunoblot analysis shows phosphorylated Met and total Met protein levels using anti‐phosphorylated Met (Tyr1234/1235) and ‐total Met antibodies. (Left lane) the branching complex of HBE135 cells in 3D culture. (Right panel) HBE135 cells treated with 10 ng/ml rHGF for 10 min. under a 2D monolayer. (B) Immunoblot analysis shows phosphorylated Met (Tyr1234/1235), total Met and Actin protein levels. Lane 1 shows HBE135 cells treated with 10 ng/ml rHGF at 10 min., Lane 2 shows HBE135 cells treated with 10 ng/ml rHGF at 10 min. after pre‐treatment with 10 ng/ml rHGF every 2 hrs (three times, total 6 hrs), and Lane 3 shows HBE135 cells treated with 10 ng/ml rHGF for 6 hrs.

**Figure S3** (A and B) Immunofluorescent images of c‐kit (green) (A) and CCSP (green)/pro‐SP‐C (red) (B) in the branching structure of HBE135 cells at 16 days in rBM. (A) Nuclear staining with PI (A) and TO‐PRO‐3 iodide (B) are shown as red and blue, respectively; scale bars: 50 μm. (C–E) Immunofluorescent images of c‐kit (C), CCSP (D) and pro‐SP‐C (E), which are shown as green, in HBE135 cells under monolayer culture. Nuclear staining with PI is shown as red. Anti‐c‐kit (A4502; DAKO) and anti‐CCSP (sc‐9772; Santa Cruz Biotechnology) antibodies were used. The cells were fixed with 4% paraformaldehyde; scale bars: 20 μm.

**Table S1** Sequences of primers used in real‐time reverse transcription ‐ PCR (RT‐PCR) assays.

## References

[1] Cardoso WV , Lü J . Regulation of early lung morphogenesis: questions, facts and controversies. Development. 2006; 133: 1611–24. 16613830 10.1242/dev.02310

[2] Butler JP , Loring SH , Patz S , *et al* Evidence for adult lung growth in humans. N Engl J Med. 2012; 367: 244–7. 22808959 10.1056/NEJMoa1203983 PMC3422892

[3] McQualter JL , Yuen K , Williams B , *et al* Evidence of an epithelial stem/progenitor cell hierarchy in the adult mouse lung. Proc Natl Acad Sci USA. 2010; 107: 1414–9. 20080639 10.1073/pnas.0909207107 PMC2824384

[4] Rock JR , Onaitis MW , Rawlins EL , *et al* Basal cells as stem cells of the mouse trachea and human airway epithelium. Proc Natl Acad Sci USA. 2009; 106: 12771–5. 19625615 10.1073/pnas.0906850106 PMC2714281

[5] Schoch KG , Lori A , Burns KA , *et al* A subset of mouse tracheal epithelial basal cells generates large colonies *in vitro* . Am J Physiol Lung Cell Mol Physiol. 2004; 286: L631–42. 12959927 10.1152/ajplung.00112.2003

[6] Delgado O , Kaisani AA , Spinola M , *et al* Multipotent capacity of immortalized human bronchial epithelial cells. PLoS ONE. 2011; 6: e22023. 21760947 10.1371/journal.pone.0022023 PMC3131301

[7] Motoi N , Szoke J , Riely GJ , *et al* Lung adenocarcinoma: modification of the 2004 WHO mixed subtype to include the major histologic subtype suggests correlations between papillary and micropapillary adenocarcinoma subtypes, EGFR mutations and gene expression analysis. Am J Surg Pathol. 2008; 32: 810–27. 18391747 10.1097/PAS.0b013e31815cb162

[8] Nakamura T , Mizuno S . The discovery of hepatocyte growth factor (HGF) and its significance for cell biology, life sciences and clinical medicine. Proc Jpn Acad Ser B Phys Biol Sci. 2010; 86: 588–610. 10.2183/pjab.86.588 PMC3081175 20551596

[9] Montesano R , Matsumoto K , Nakamura T , *et al* Identification of a fibroblast‐derived epithelial morphogen as hepatocyte growth factor. Cell. 1991; 67: 901–8. 1835669 10.1016/0092-8674(91)90363-4

[10] Mizuno S , Ohnishi H , Nakamura T . Hepatocyte growth factor (HGF), and endogenous pulmotrophic regulator, for the rescue of acute and chronic lung disease. Curr Signal Transd Ther. 2011; 6: 210–20.

[11] Sakamaki Y , Matsumoto K , Mizuno S , *et al* Hepatocyte growth factor stimulates proliferation of respiratory epithelial cells during postpneumonectomy compensatory lung growth in mice. Am J Respir Cell Mol Biol. 2002; 26: 525–33. 11970903 10.1165/ajrcmb.26.5.4714

[12] Shigemura N , Sawa Y , Mizuno S , *et al* Amelioration of pulmonary emphysema by *in vivo* gene transfection with hepatocyte growth factor in rats. Circulation. 2005; 111: 1407–14. 15781752 10.1161/01.CIR.0000158433.89103.85

[13] Yamamoto H , Yun EJ , Gerber HP , *et al* Epithelial‐vascular cross talk mediated by VEGF‐A and HGF signaling directs primary septae formation during distal lung morphogenesis. Dev Biol. 2007; 308: 44–53. 17583691 10.1016/j.ydbio.2007.04.042

[14] Zhou Z , Hao Y , Raptis L , *et al* TAZ is a novel oncogene in non‐small cell lung cancer. Oncogene. 2011; 30: 2181–6. 21258416 10.1038/onc.2010.606

[15] Kuo PL , Hsu YL , Huang MS , *et al* Ginger suppresses phthalate ester‐induced airway remodeling. J Agric Food Chem. 2011; 59: 3429–38. 21370925 10.1021/jf1049485

[16] Kato T , Funakoshi H , Kadoyama K , *et al* Hepatocyte growth factor overexpression in the nervous system enhances learning and memory performance in mice. J Neurosci Res. 2012; 90: 1743–55. 22535512 10.1002/jnr.23065

[17] Nakamura Y , Niki T , Goto A , *et al.* c‐Met activation in lung adenocarcinoma tissues: an immunohistochemical analysis. Cancer Sci. 2007; 98: 1006–13. 17459054 10.1111/j.1349-7006.2007.00493.x PMC11159971

[18] Franzdóttir SR , Axelsson IT , Arason AJ , *et al* Airway branching morphogenesis in three dimensional culture. Respir Res. 2010; 11: 162. 21108827 10.1186/1465-9921-11-162 PMC3002372

[19] Wansleeben C , Barkauskas CE , Rock JR , *et al* Stem cells of the adult lung: their development and role in homeostasis, regeneration, and disease. Wiley Interdiscip Rev Dev Biol. 2013; 2: 131–48. 23799633 10.1002/wdev.58

[20] Kapanci Y , Weibel ER , Kaplan HP , *et al* Pathogenesis and reversibility of the pulmonary lesions of oxygen toxicity in monkeys. II. Ultrastructural and morphometric studies. Lab Invest. 1969; 20: 101–18. 4988417

[21] Evans MJ , Cabral LJ , Stephens RJ , *et al* Renewal of alveolar epithelium in the rat following exposure to NO_2_ . Am J Pathol. 1973; 70: 175–98. 4566990 PMC1903972

[22] Feng Y , Thiagarajan PS , Ma PC . MET signaling: novel targeted inhibition and its clinical development in lung cancer. J Thorac Oncol. 2012; 7: 459–67. 22237263 10.1097/JTO.0b013e3182417e44

[23] Ohmichi H , Koshimizu U , Matsumoto K , *et al* Hepatocyte growth factor (HGF) acts as a mesenchyme‐derived morphogenic factor during fetal lung development. Development. 1998; 125: 1315–24. 9477330 10.1242/dev.125.7.1315

[24] Volckaert T , De Langhe S . Lung epithelial stem cells and their niches: Fgf10 takes center stage. Fibrogenesis Tissue Repair. 2014; 7: 8. 24891877 10.1186/1755-1536-7-8 PMC4041638

[25] Kajstura J , Rota M , Hall SR , *et al* Evidence for human lung stem cells. N Engl J Med. 2011; 364: 1795–806. 21561345 10.1056/NEJMoa1101324 PMC3197695

[26] Kim CF , Jackson EL , Woolfenden AE , *et al* Identification of bronchioalveolar stem cells in normal lung and lung cancer. Cell. 2005; 121: 823–35. 15960971 10.1016/j.cell.2005.03.032

[27] Tsao MS , Liu N , Chen JR , *et al* Differential expression of Met/hepatocyte growth factor receptor in subtypes of non‐small cell lung cancers. Lung Cancer. 1998; 20: 1–16. 9699182 10.1016/s0169-5002(98)00007-5

[28] Nakamura T , Sakai K , Nakamura T , *et al* Hepatocyte growth factor twenty years on: much more than a growth factor. J Gastroenterol Hepatol. 2011; 26: 188–202. 21199531 10.1111/j.1440-1746.2010.06549.x

