# Supplementary figures and images for: Bronchioalveolar morphogenesis of human bronchial epithelial cells depending upon hepatocyte growth factor
Source: J Cell Mol Med. 2015 Sep 28;19(12):2818–26. doi: 10.1111/jcmm.12672 (PMC4687712; doi:10.1111/jcmm.12672)

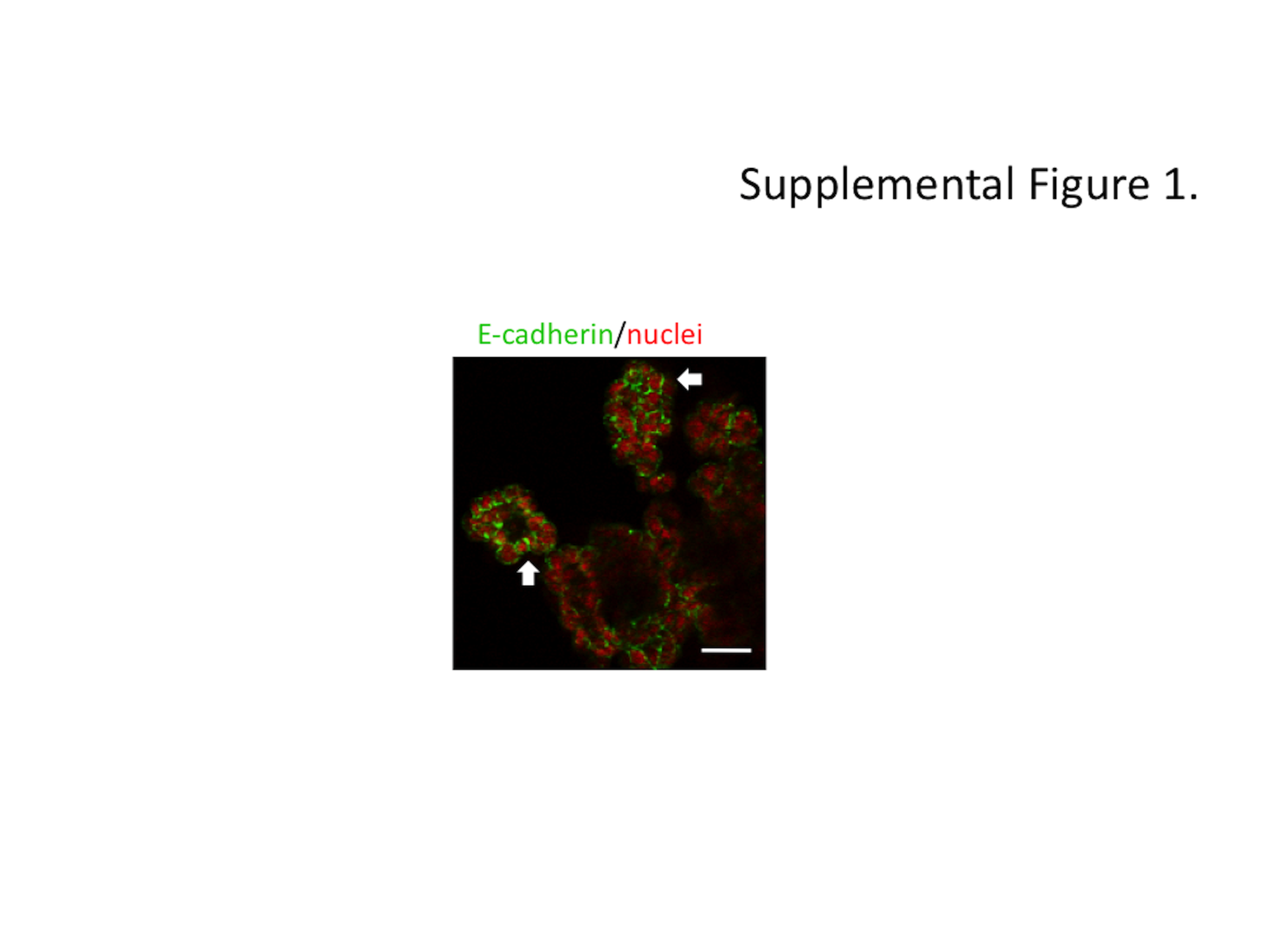

Supplement: Supplementary file 1 — Figure S1 Higher magnification of image for E‐cadherin in the branching structure co‐cultured with MRC‐9 cells in Matrigel. White arrows indicate that the E‐cadherin was stained stronger at cell–cell junctions; scale bars: 20 μm. [file JCMM-19-2818-s001.tif]

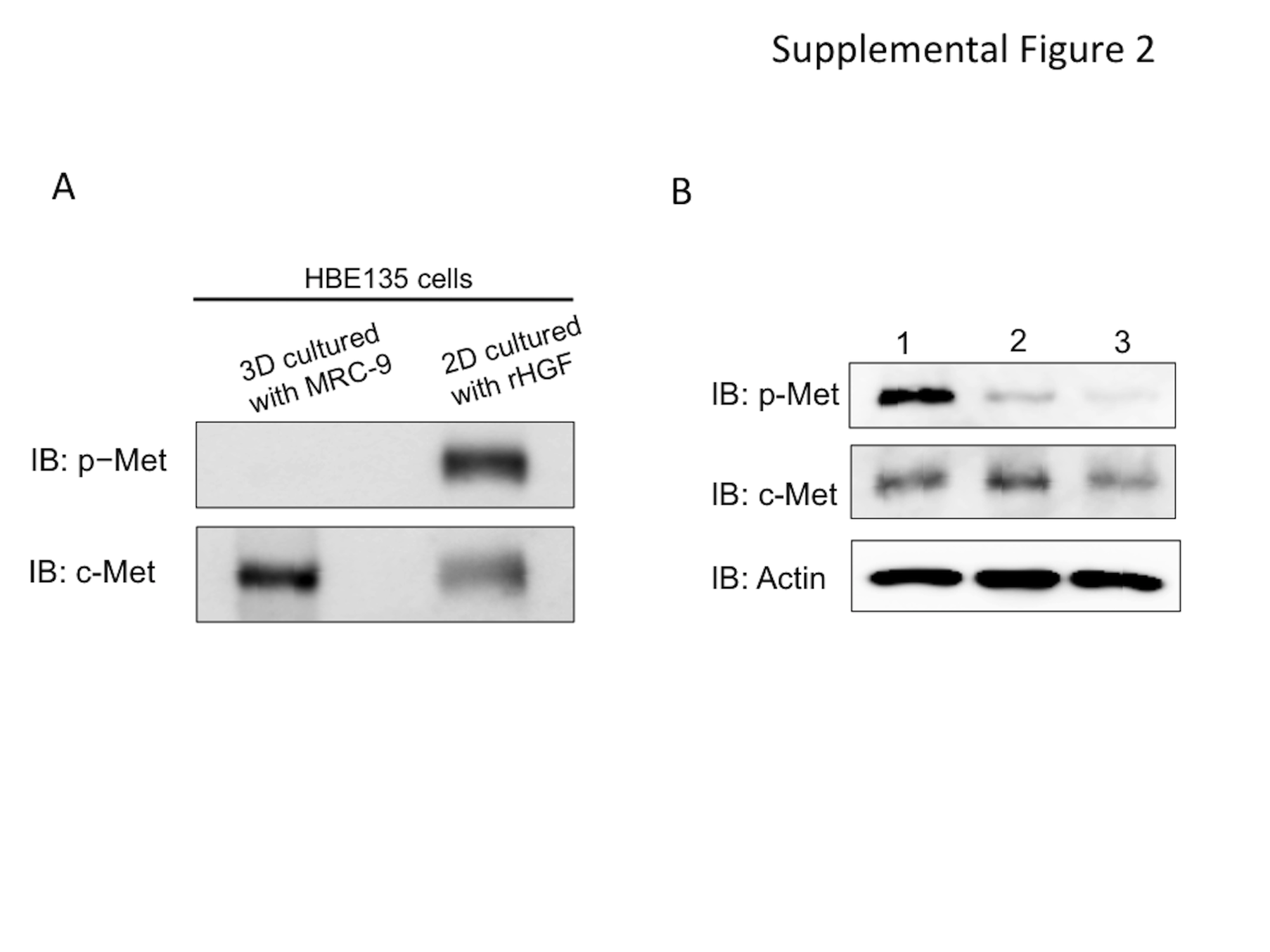

Supplement: Supplementary file 2 — Figure S2 (A) Met of the branching structure was not phosphorylated in 3D co‐culture with MRC‐9 cells. Immunoblot analysis shows phosphorylated Met and total Met protein levels using anti‐phosphorylated Met (Tyr1234/1235) and ‐total Met antibodies. (Left lane) the branching complex of HBE135 cells in 3D culture. (Right panel) HBE135 cells treated with 10 ng/ml rHGF for 10 min. under a 2D monolayer. (B) Immunoblot analysis shows phosphorylated Met (Tyr1234/1235), total Met and Actin protein levels. Lane 1 shows HBE135 cells treated with 10 ng/ml rHGF at 10 min., Lane 2 shows HBE135 cells treated with 10 ng/ml rHGF at 10 min. after pre‐treatment with 10 ng/ml rHGF every 2 hrs (three times, total 6 hrs), and Lane 3 shows HBE135 cells treated with 10 ng/ml rHGF for 6 hrs. [file JCMM-19-2818-s002.tif]

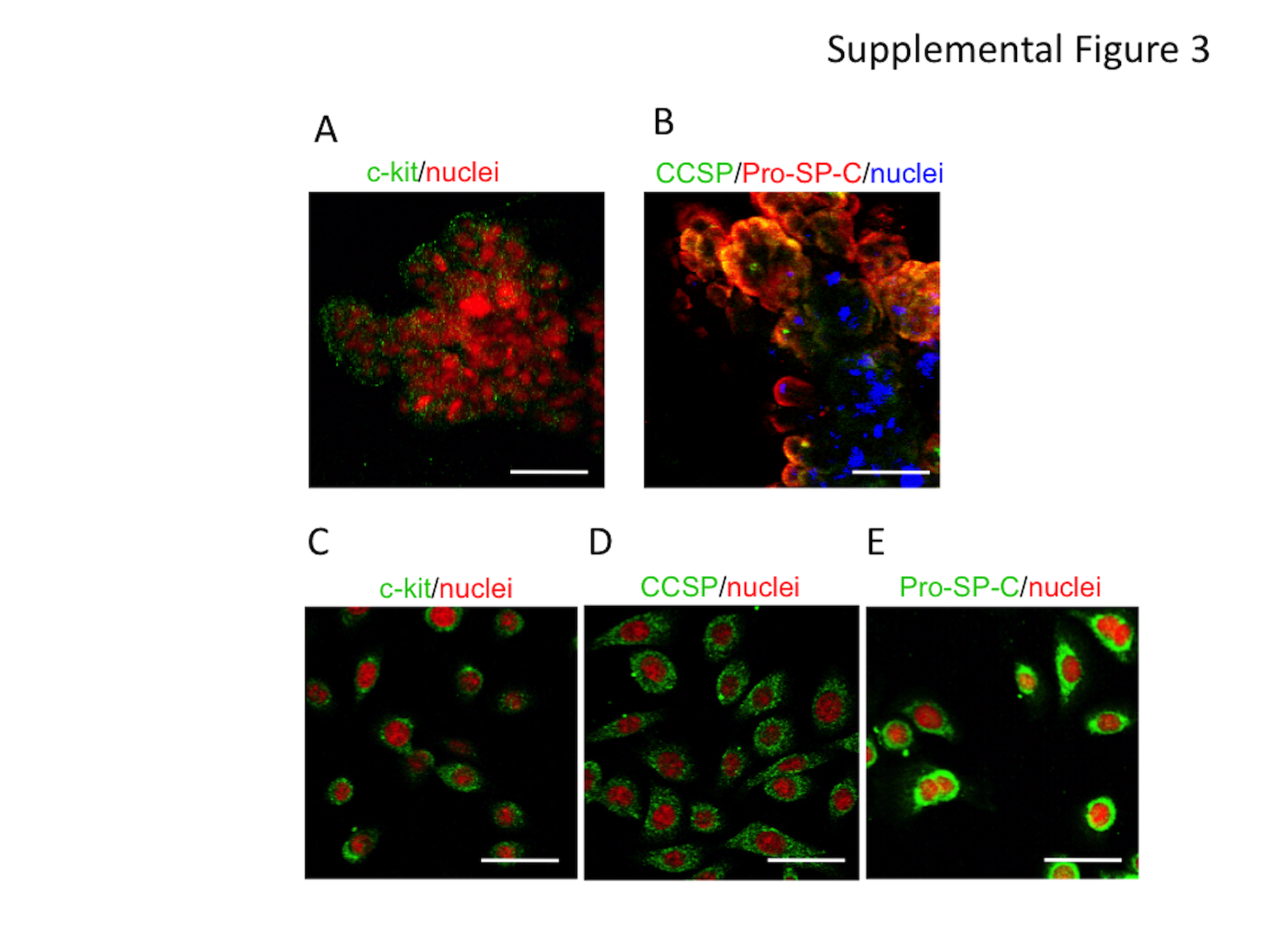

Supplement: Supplementary file 3 — Figure S3 (A and B) Immunofluorescent images of c‐kit (green) (A) and CCSP (green)/pro‐SP‐C (red) (B) in the branching structure of HBE135 cells at 16 days in rBM. (A) Nuclear staining with PI (A) and TO‐PRO‐3 iodide (B) are shown as red and blue, respectively; scale bars: 50 μm. (C–E) Immunofluorescent images of c‐kit (C), CCSP (D) and pro‐SP‐C (E), which are shown as green, in HBE135 cells under monolayer culture. Nuclear staining with PI is shown as red. Anti‐c‐kit (A4502; DAKO) and anti‐CCSP (sc‐9772; Santa Cruz Biotechnology) antibodies were used. The cells were fixed with 4% paraformaldehyde; scale bars: 20 μm. [file JCMM-19-2818-s003.tif]
