# Supplementary material for: Bronchioalveolar morphogenesis of human bronchial epithelial cells depending upon hepatocyte growth factor
Source: J Cell Mol Med. 2015 Sep 28;19(12):2818–26. doi: 10.1111/jcmm.12672 (PMC4687712; doi:10.1111/jcmm.12672)
Supplement: Supplementary file 4 — Table S1 Sequences of primers used in real‐time reverse transcription ‐ PCR (RT‐PCR) assays. [file JCMM-19-2818-s004.doc]

Supplemental Table1

| **Transcript** |  | **Sequence** |
| --- | --- | --- |
| FGF1 |  | F: 5'-AGTACTTGGCCATGGACACC-3' |
|  |  | R: 5'-CTTCTTGAGGCCAACAAACC-3' |
| FGF7 |  | F: 5'-CATGGAAATCAGGACAGTGG-3' |
|  |  | R: 5'-TTCCCCTCCGTTGTGTGTCC-3' |
| HGF |  | F: 5'-TTCCAACACGAACAAACATAGG-3' |
|  |  | R: 5'-AGTCTCGAGAAGGGAAACACTG-3' |
| HB-EGF |  | F: 5'-TGGGGCTTCTCATGTTTAGG-3' |
|  |  | R: 5'-CATGCCCAACTTCACTTTCTC-3' |
| VEGF-A |  | F: 5'-AAGGAGGAGGGCAGAATCAT-3' |
|  |  | R: 5'-ATCTGCATGGTGATGTTGGA-3' |
| Transcript and sequence of each primer used in real time RT-PCR. | | |
| In the table, F indicates forward primer, R indicates reverse primer. | | |
